# Metabolically Healthy Obesity and Risk of Incident Chronic Kidney Disease in a Korean Cohort Study

**Published:** 2019-11

**Authors:** Youngran YANG

**Affiliations:** Research Institute of Nursing Science, School of Nursing, Jeonbuk National University, Jeonbuk, Republic of Korea

**Keywords:** Metabolically healthy obesity, Chronic kidney disease, Obesity, Metabolic syndrome, Risk

## Abstract

**Background:**

The incident of chronic kidney disease (CKD) of metabolically healthy obesity (MHO) has not been consistently determined.

**Methods:**

This study used data of Anseong Ansan community-based cohort, a part of the Korean Genome and Epidemiology Study (KoGES) provided by the Korea Center for Disease Control and Prevention (KCDC). Surveys were received from the Anseung and Ansan residents every two years between 2001–2002 and 2015–2016 for a total of 7 surveys over all. The subjects were divided into 4 phenotypes based on the presenting obesity and metabolic syndrome; 1) metabolically healthy normal weight (MHNW), 2) metabolically healthy obesity (MHO), 3) metabolically abnormal normal weight (MANW), and 4) metabolically abnormal obesity (MAO). Data were analyzed using the Cox proportional hazards regression model.

**Results:**

Of 8,865 subjects, 1,551 cases of 49,995 person-year (3.1%) developed incident CKD. At an adjusted hazard ratio (HR) of 1.13, the MHO group was not associated with a higher risk of incident CKD (95% confidence interval (CI): 0.92–1.41, *P* =0.234, using MHNW as the reference). The adjusted HRs of the MANW and MAO groups for incident CKD were significantly higher than those of the MHNW groups: 1.31 (95% CI: 1.05–1.64, *P*=0.017) for MANW and 1.49 (95% CI: 1.23–1.79, *P*<0.001) for MAO.

**Conclusion:**

MHO is not associated with a high risk of CKD, and that MANW and MAO increase the risk of the incident CKD. Thus, it is important to consider metabolic health status rather than obesity when evaluating CKD risk.

## Introduction

Chronic kidney disease (CKD) is associated with loss of kidney function and other complications. Worldwide, the estimated mean prevalence of CKD was 10.6% among 6,908,440 patients in a meta-analysis of 5,842 potential articles (1). CKD has become a substantial public health burden with high medical expenses and decreased quality of life (2, 3). The attendant socio-economic costs (medical expenses, nursing care, time cost, death loss, disability loss) due to CKD are estimated at 5.2 million USD in 2011, and the social burden is expected to increase steadily (2).

“Metabolically healthy obesity (MHO)" is recently attracting attention (4). MHO is a clinical expression that refers to a metabolic healthy state due to relatively low levels of insulin sensitivity, visceral obesity, blood pressure and lipid metabolism. Although MHO is metabolically healthy, the long-term prevalence of cardiovascular disease, diabetes, and cancer and even mortality rates are high in this group (5, 6). However, whether MHO is a risk factor for CKD has not been elucidated, thus establishing a need to investigate the relationship between MHO and CKD (7).

Few studies conducted have not shown consistent results. In a study among 3,136 Japanese people using a 8-year follow-up survey of work-place health screenings, those in the MHO group were more likely to develop CKD than those determined to be metabolically healthy and non-obese (odds ratio (OR): 0.83, 95% confidence interval (CI): 0.36–1.72, *P* = 0.64) (8). A cross-sectional study on 2,324 Chinese people found that the MHO group did not have a higher risk of CKD (OR: 0.79, 95% CI: 0.29–2.14, *P*=0.64) (9). Consistent with the result of that Chinese study was the outcome of the Tehran Lipid Glucose Study in Iranian study (2015) (hazard ratio (HR): 1.23, 95% CI: 0.93–1.62) (10). In a Korean study, in the MHO group 1.38 times more likely to develop CKD than the MHNW group (HR: 1.38, 95% CI: 1.01–1.87) (11). In addition, these existing studies have multiple limitations: short follow-up periods that not is sufficient to elucidate the risk of CKD (8), cross-sectional study designs that are unable to identify cause-and-effect relationships (9), and a lack of reflection on the effects of lifestyle choices like exercising, smoking, and drinking (8). The general population was not represented (11). This study was designed to overcome these all limitations.

The aim of this study was to determine the long-term relationship between MHO and CKD. This study was designed to specifically address the limitations of previous studies by using 14 years of long-term follow-up data; quantitatively measuring lifestyle habits like exercising, smoking, and drinking; and using data that represents the general adult population of Korea.

## Materials and Methods

### Data and study population

This study used data of Anseong Ansan community-based cohort, a part of the Korean Genome and Epidemiology Study (KoGES) provided by the Korea Center for Disease Control and Prevention (KCDC). Surveys were received from the Anseung and Ansan residents every two years between 2001–2002 and 2015–2016 for a total of 7 surveys over all.

The data of the 10,030 subjects (5,018 from Anseung and 5,012 from Ansan) were reviewed originally for analysis and those meeting the following conditions are excluded: 1) GFR <60 ml / min per 1.73 m^2^ (equivalent to 3–5 levels of CKD) (CKD baseline n=226 plus proteinuria n=251); 2) history of cancer (n= 82); 3) kidney disease and/or urinary tract infection (n= 326); and 4) missing data at baseline survey (n=280). In the end, 8,865 participants were included in the analysis.

### Ethical approval

Institutional Review Board at the Korea Center for Disease Control and Prevention (KCDC) and Jeonbuk National University

### Definition of metabolic health and obesity states

Metabolic syndrome was defined by the National Cholesterol Education Program Adult Treatment Panel III (NCEP-ATP) (13). Obesity in this study was defined using the Asian standard for BMI, which is 25 kg/m^2^ or more (11). Subjects were divided into phenotypes according to the combination of the presence or absence of obesity and the presence or absence of metabolic syndrome: 1) MHNW;(2) MHO;(3) metabolic abnormal, normal weight (MANW); and (4) metabolic abnormal, obesity (MAO).

### Determination of incident CKD

CKD is defined as a situation where a person’s glomerular filtration rate (GFR) is less than 60/min per 1.73 m^2^. Since there was no GFR value in this integrated data, serum creatinine levels were used to calculate the GFR value using the formula of CKD Epidemiology Collaboration (CKD-EPI): GFR=141× min (Scr/ϰ,1)^α^ × max (Scr/ϰ,1)^−1.209^ × 0.993^Age^ × 1.018 (female), where Scr is serum creatinine (mg/dl), ϰ is 0.7 (female) or 0.9 (male), α is 0.329 (female) or –0.411 (male), and *min* and *max* are the minimum and maximum values of Scr/ϰ (14).

### Clinical and laboratory measurements

For both systolic and diastolic blood pressure, three measurements were taken and the mean was used. The cohort provided data on total alcohol consumption (g/d), total tobacco use (pack/yr), and total physical activity (metabolic equivalent of task, MET/wk).

### Statistical analysis

SPSS 20.0 (Chicago, IL, USA) and R programming (for calculating GFR) were used for data processing and analysis. The life table analysis that shows the curve for cumulative survival free from incident CKD of 4 phenotypes of obesity was used. The hazard ratio was calculated using the Cox proportional hazards regression model to compare the risk of CKD according to the 4 groups. MHNW was set as the reference group; the hazard ratio and the 95% CI of MHO, MANW and MAO were obtained to determine the statistical significance. Three models were run after adjusting for variables: age, sex, and income in Model 1, plus baseline GFR, drinking, smoking, physical activity, and history of cardiovascular disease for Model 2, plus ALT, AST, uric acid, CRP, GTP, and systolic blood pressure for Model 3.

### Results

Of the participants, 70.6% (n=6,256) were metabolically healthy and, of these, 42.3% (n=3,747) were obese. The MHO group accounted for 21.5% (1,902) of the total subjects and 50.8% of the obese population. Compared to the MHNW subjects, those in the MHO group were more likely to be male, have a higher income and engage in physical activity less. During the 14-year follow-up period, 49,995 person-years, which is the number of follow-up times multiplied by the number of people, were determined, and 1,551 cases of the person-year (3.1%) developed incident CKD (Table 1).

**Table 1:** Baseline clinical and biochemical characteristics of study subjects according to metabolic health and obesity

***Variables***	***Normal weight (BMI<25 kg/m^2^)***		***Obese (BMI ≥25 kg/m^2^)***		
	***Metabolically healthy (MHNW)^a^ (n=4,354)***	***Metabolically abnormal (MANW)^b^ (n=764)***	***Metabolically healthy (MHO) (n=1,902)***	***Metabolically abnormal (MAO)^d^ (n=1,845)***	**P *for trend***
Number of person-years	2,4771.5	4,036.5	11,138	10,049	
Number of incident CKD	615	205	266	465	
Incidence rate(per 10,000 person-years)	248	508	239	463	<0.001
Age(years) (Mean±SD)	51.1±8.9	56.3±8.6	49.6±7.8	53.6±8.4	<0.001
Male (%)	48.0	56.7	53.8	56.9	<0.001
Household income/month, in won (more than 2,000 USD, %)^1)^	37.9	27.0	44.7	32.6	<0.001
Education (%)					<0.001
Elementary	28.0	47.6	25.8	42.1	
Middle	23.2	20.4	24.7	22.1	
High	34.5	20.9	32.4	24.3	
University	14.3	11.0	17.1	11.6	
Marital Status (%)					<0.001
Single	1.6	0.9	0.9	1.2	
Married	90.6	85.3	91.0	87.2	
Widowed/divorced/separated	7.9	13.7	8.1	11.6	
BMI(kg/m^2^) (Mean±SD)	22.3±1.9	23.2±1.5	26.9±1.8	27.9±2.3	<0.001
Waist circumference(cm)	77.0±6.5	83.2±6.6	85.5±6.4	91.8±6.1	<0.001
History of CVD (%)	1.9	3.3	2.1	3.5	<0.001
Total alcohol(g/day)	10.0±22.6	9.1±22.2	9.8±21.1	9.7±23.0	0.754
Smoking(pack/year)	10.3±16.3	10.5±17.5	7.9±14.5	8.9±15.5	<0.001
Physical activity(MET/week)	9760.6±6423.0	10078.3±7030.1	9056.3±5773.5	9537.9±6473.7	<0.001
Systolic BP(mmHg)	116.0±16.8	132.1±17.6	117.2±15.6	132.0±17.5	<0.001
Diastolic BP(mmHg)	76.7±10.6	85.8±10.6	78.6±10.5	87.2±10.4	<0.001
eGFR(mL/min/1.73m^2^)	94.1±13.0	91.5±12.1	92.9±13.7	91.0±12.8	<0.001
Fasting blood glucose(mg/dl)	83.3±15.0	95.5±35.2	84.8±16.9	94.1±24.7	<0.001
Triglycerides(mg/dl)	129.3±76.9	224.2±112.0	141.2±77.5	224.7±128.7	<0.001
HDL cholesterol(mg/dl)	53.2±12.3	43.5±9.3	50.2±11.2	43.5±8.6	<0.001
LDL cholesterol(mg/dl)	115.0±33.0	113.7±36.4	125.2±32.9	121.2±36.4	<0.001
HOMA-IR((uU/ml, mg/dl)	24.7±21.1	34.1±27.0	29.9±17.5	40.1±26.1	<0.001
ALT(IU/L)	24.7±20.3	30.1±31.3	29.1±39.1	34.0±22.3	<0.001
AST(IU/L)	29.0±16.7	31.4±23.7	29.1±20.8	31.0±14.0	<0.001
CRP(mg/dl)	0.2±0.5	0.3±0.9	0.2±0.4	0.3±0.3	<0.001
GTP(IU/L)	31.8±69.4	48.6±106.0	31.4±38.0	41.5±50.0	<0.001
Uric acid(mg/dl)	45.3±21.3	41.4±20.1	47.1±22.6	45.5±20.7	<0.001

*Note.* Data are expressed as percentage (number) or Mean±SD *1)* $1 USD≒1000 won, BMI: body mass index, CVD history: chronic heart failure, coronary artery disease, myocardial infarction, cerebrovascular accident, 3)MET: metabolic equivalent of task, alcohol intake by alcohol(g/month) = frequency (times/month × amount of cup/time × alcohol g/cup, Total alcohol consumption (g/day) = [alcohol intake by alcohol(g/month) / 30, alcohol g/cup (g) × amount /cup (cc) × Ethanol gravity (0.7893) × alcohol degree (%) /100, HDL-C: high density lipoprotein cholesterol, HOMA-IR: homeostatic model assessment-insulin resistance, ALT: alanine aminotransferase, AST: aspartate aminotransferase, CRP: C-reactive protein, GTP: gamma-glutamyltransferase

The crude incidence rate was 2.5% (615/24771.5 person-year) in the MHNW group, 2.4% (266/11,138 person-year) in the MHO, 5.1% (205/4,036.5 person-year) in the MANW group, and 4.5% (465/10,049 person-year) in the MAO group.

**Fig. 1:**
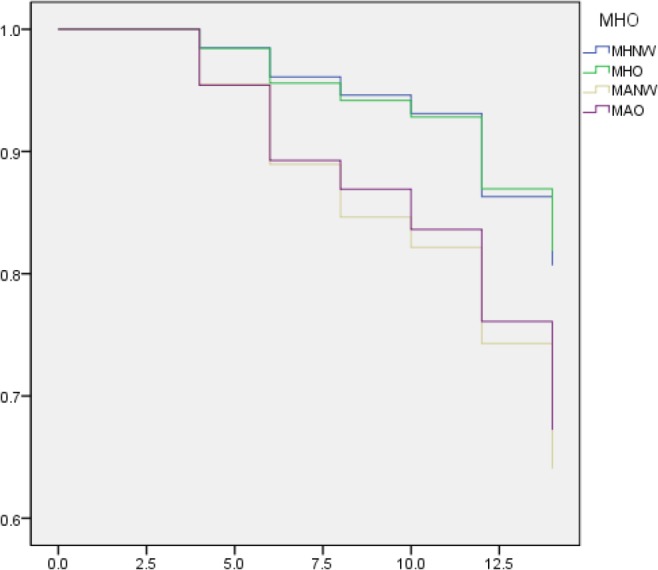
CKD-free survival determination according to Metabolic Health and Obesity

The curve for cumulative survival free from incident CKD is presented in Fig. 1. Those in the MANW and MAO groups had a higher probability of developing incident CKD than those in the MHNW and MHO groups, but within these groups, there were no significant differences between MHNW and MHO or MANW and MAO individuals (log-rank test, *P* =0.642, *P* =0.299, respectively).

Table 2 shows the associations of obesity categories (non-obese, obese), BMI (under, normal, over, obese), NECA ATP-III components and numbers, and the results of running Model 1, Model 2, and Model 3. The adjusted hazard ratio (HR) of obese individuals for incident CKD (using non-obese subjects as the reference) was 1.22 (95% CI: 1.06–1.42, *P*=0.006, Model 3). The adjusted hazard ratio (HR) of underweight individuals was 0.48 (95% CI: 0.25–0.91, *P*=0.023, Model 3), and the adjusted HR of obese individuals for incident CKD was 1.24 (95% CI: 1.03–1.48, *P*=0.021, Model 3, with normal weight used as the reference). All ATP-III components were associated with incident CKD in Model 1 and Model 2, with HRs ranging from 1.14 to 1.44. In Model 3, the adjusted HR was 1.33 (95% CI: 1.15–1.54, *P<*0.001) for high triglyceride levels and 1.36 (95% CI: 1.14–1.63, *P*=0.001) for high blood plasma glucose, respectively. As the number of metabolic syndrome components increased from 2 to 5, the risk of CKD increased proportionally from 1.38 (95% CI: 1.14–1.67, *P*=0.001) to 2.35 (95% CI: 1.75–3.15, *P<*0.001, Model 2).

**Table 2:** Hazard ratios for the development of incident CKD according to Obesity, ATP-III components, metabolic health and Metabolic Health and Obesity status

***Variable***	***Model 1***	***Model 2 ***	***Model 3***
Obesity			
Non-obese (BMI<25)	1.0 (ref.)	1.0 (ref.)	1.0 (ref.)
Obese(BM≥25)	1.27 (1.15–1.40)	1.17 (1.06–1.30)	1.22 (1.06–1.42)
BMI			
Normal(18.5–22.9)	1.0 (ref.)	1.0 (ref.)	1.0 (ref.)
Under(>18.5)	0.68 (0.45–1.04)	0.65 (0.42–0.99)	0.48 (0.25–0.91)
Overweight(23–24.9)	1.16 (1.01–1.33)	1.10 (0.95–1.27)	1.06 (0.87–1.30)
Obese(≥25)	1.34 (1.19–1.52)	1.21 (1.07–1.37)	1.24 (1.03–1.48)
NCEP-ATP III Components			
WC	1.32 (1.19–1.46)	1.27 (1.14–1.41)	1.10 (0.95–1.28)
TG	1.14 (1.28–1.56)	1.44 (1.30–1.60)	1.33 (1.15–1.54)
HDL-C	1.31 (1.17–1.46)	1.32 (1.18–1.48)	1.15 (0.98–1.34)
FPG or on medication	1.33 (1.17–1.51)	1.33 (1.17–1.52)	1.36 (1.14–1.63)
BP or on medication	1.24 (1.18–1.38)	1.30 (1.17–1.45)	1.10 (0.90–1.36)
Number of NCEP ATP-III components			
0	1.0 (ref.)	1.0 (ref.)	1.0 (ref.)
1	1.12 (0.93–1.36)	1.13 (0.93–1.37)	0.93 (0.70–1.24)
2	1.46 (1.22–1.75)	1.38 (1.14–1.67)	1.08 (0.82–1.42)
3	1.61 (1.33–1.95)	1.65 (1.36–2.01)	1.32 (0.99–1.75)
4	1.97 (1.61–2.41)	2.00 (1.63–2.46)	1.44 (1.05–1.97)
5	2.37 (1.78–3.15)	2.35 (1.75–3.15)	1.79 (1.18–2.70)
Metabolic health			
Metabolically health	1.0 (ref.)	1.0 (ref.)	1.0 (ref.)
Metabolically unhealthy	1.45 (1.30–1.60)	1.51 (1.36–1.68)	1.36 (1.17–1.60)
Metabolic Health and Obesity status			
MHNW	1.0 (ref.)	1.0 (ref.)	1.0 (ref.)
MHO	1.10 (0.95–1.26)	0.97 (0.83–1.12)	1.13 (0.92–1.41)
MANW	1.34 (1.14–1.57)	1.41 (1.20–1.66)	1.31 (1.05–1.64)
MAO	1.56 (1.38–1.76)	1.53 (1.35–1.74)	1.49 (1.23–1.79)

Abbreviations: NCEP ATP-III, National Cholesterol Education Program Adult Treatment Panel-III; BMI, body mass index; BP, blood pressure; CKD, chronic kidney disease; FPG, fasting plasma glucose or on medication; HDLC, high density lipoprotein cholesterol; ref, reference; TG, triglyceride

Model 1 was adjusted for age, sex, and income

Model 2 was adjusted for the variables in model 1, plus baseline glomerular filtration rate (GFR), drinking, smoking, physical activity, history of cardiovascular disease (CVD)

Model 3 was adjusted for the variables in model 2, plus alanine aminotransferase (ALT), Aspartate aminotransferase (AST), uric acid, C-reactive protein (CRP), gamma-glutamyltransferase (GTP), systolic blood pressure

The HRs significantly remained in Model 3, 1.44 times when 4 components were present (95% CI: 1.05–1.97, *P*=0.034) and 1.79 times when 5 components were present (95% CI: 1.18–2.70, *P*=0.006).

Compared to metabolically healthy individuals (irrespective of obesity), metabolically unhealthy individuals had 1.36 (95% CI: 1.17–1.60, *P<*0.001) times the risk of developing CKD. The adjusted HRs of MANW subjects (1.31, 95% CI: 1.05–1.64, *P*=0.017) and MAO subjects (1.49, 95% CI: 1.23–1.79, *P*=<0.001) for incident CKD were also significantly higher than those of the MHNW group. The HRs of MHO subjects were not significant in any Models (Table 2).

## Discussion

The results of this study showed that MHO was not a risk for incident CKD, which was consistent with the results of prospective cohort studies in the people of Japan (8) and Iran (10). MHO was associated with CKD development (RR= 1.235, 95% CI: 1.027, 1.484) (15) suggesting that MHO status may be involved in CKD incidence. The high risk relationship between MHO and CKD is partly explained by the role of chronic inflammation as measured by hsCRP level; the risk of developing CKD among all four obesity phenotypes (MHNW, MHO, MANW and MAO) was higher for individuals with hsCRP ≥ 0.20 mg/L than hsCRP < 0.20 mg/L (16). The associations were stronger for those subjects identified as metabolically abnormal (ORs ranging from 3.43 to 4.61) than those considered metabolically healthy (ORs ranging from 2.01 to 2.91). With the addition of hsCRP in the model, while the odds risk of MHO was no longer significant (OR: 1.52, 95% CI: 0.93, 2.49), the ORs were still maintained for MANW and MAO (16). Those factors strongly support the idea that systematic inflammation is independently involved in CKD in addition to the effect of metabolic health status irrespective of obesity.

As shown by the significantly higher HRs of obese groups reported in Table 2, this study supports other studies indicating obesity as a risk factor for the onset and progression of CKD when metabolic health was not considered (17). Increased visceral adiposity, fatty acids, cytokines, and adipokines may cause a decline in kidney function, leading to the development of hypertension (18), and obesity itself could be harmful to renal function (19). These results suggest that metabolic syndrome is a bigger risk factor than weight for the development of CKD (Fig. 1). In one meta-analysis (20), the risk of CKD was much higher in metabolically unhealthy groups regardless of obesity condition; risk ratio was 1.572 (95% CI: 1.373, 1.801) for MANW and 1.898 (95% CI: 1.505, 2.395) for MAO compared to MHNW, indicating that one may not be free from the risk of CKD if one has a normal weight but an unhealthy metabolic condition. In another study (20), waist circumstance (WC) and waist-to-height ratio (WHtR) were more closely related to CKD than BMI.

WHtR is a better indicator for measurement for obesity in men than BMI, and WC in women is a better indicator in relation to CKD. Future studies should consider various indicators of obesity when studying the long-term risk of CKD in metabolically healthy obesity, or look at relationships among obesity, metabolic syndrome, and CKD.

In all models, the risk of CKD was 1.36 ~ 1.51 times higher in the metabolically unhealthy group than the healthy group. Having two factors of a metabolic syndrome component without a diagnosis of metabolic syndrome did not preclude CKD. Overall, the incidence of CKD increased proportionally with the number of metabolic syndrome components: 1.44 times HR for CKD development in the 4 components group and 1.79 times HR in the 5 components group. Metabolic syndrome was a crucial risk factor for CKD development and the risks were increased by the number of components of metabolic syndrome that are presenting (21–23). The odds ratio was 1.744 (CI 95%: 1.296, 2.347) when 4 components of metabolic syndrome were present and 2.109 (CI 95%: 1.219, 3.651) in the case of 5 components (22). In a meta-analysis of metabolic syndrome and kidney disease, the association was bigger proportionally as the number of components of metabolic syndrome increased from 2 to 5; ORs were 1.39, 1.42, 1.66, and 1.96, with increasing increments of 0.03, 0.24, and 0.3 (24). Another study had a risk ratio (RR) of CKD that was higher than that of the meta-analysis; having one component (risk ratio, RR 1.49, 95% CI: 1.10, 2.01), two components (RR 1.89, 95% CI: 1.38, 2.59), more than three components (RR 2.65, 95% CI: 1.91, 3.68) (23). Thus, based on the results of both previous research and this study, having one or two components could be a risk factor for CKD despite the presence or absence of metabolic syndrome, and the risk becomes proportionally bigger as the number of components grows. Because of this, early detection and treatment of metabolic syndrome should be strengthened by CKD prevention strategies. In addition, for healthy people who are not diagnosed with metabolic syndrome, screening for CKD should become more common at public health centers. Since the presence of even 1 component during screening can indicate a risk for CKD, it would be good to assess for kidney function at this time. This study also found an association between a higher risk of CKD and high triglyceride levels (HR 1.33) and high fasting plasma glucose levels (HR 1.36). A meta-analysis reported the RR of each component for CKD incidence; higher blood pressure (RR 1.61), lower HDL cholesterol (RR 1.23), high triglyceride levels (RR 1.27), and abdominal obesity (RR 1.19), hyperglycemia (RR 1.14) (24). Among Korean subjects, the CKD risk factor was 2.47 times higher for impaired fasting glucose than normal fasting glucose and 2.42 times higher for high triglyceride levels compared to normal ones (25). Past research has shown that elevated metabolized triglyceride-rich apoB-containing lipoproteins may promote the progression of renal insufficiency (26). Therefore, individuals who have high triglyceride and blood sugar levels should be carefully monitored to prevent CKD and educated to manage their triglycerides and blood sugar even if they are not diagnosed with metabolic syndrome. It is also necessary to study how hyperlipidemia and hyperglycemia increase the incidence of CKD. Among the components of metabolic syndrome, hypertension, abdominal obesity, and low HDL cholesterol are associated with CKD, and their mechanism has been confirmed in meta-analysis and other studies (24, 27). However, in this study, hypertension, lower HDL cholesterol and abdominal obesity were not related to CKD development, suggesting the need for further investigation.

This study has some limitations. First, GFR values were estimated using the CKD-EPI formula based on creatinine rather than actual GFR measurements, which could overestimate or underestimate the GFR. Second, 4 different types of obesity and metabolic health state were classified based on baseline in the first year, so the classifications could be shifted throughout 14 years of follow-ups. Third, this study did not use central obesity such as WC or WHtR, which is more representative of adipose tissue distribution and more associated with CKD. Future studies should address these limitations.

This study also had several strengths. First, this can be considered as a representative study of Koreans using data sampled in accordance with a standardized process in the country and followed by 14 years of follow-up. Lifestyle was more precisely and accurately reflected in the analysis by using total alcohol consumption (g / d), smoking (pack / yr) and physical activity (MET/wk) instead of simply using frequency or yes / no questions to determine usage or engagement. Finally, the KoGES surveys provided detailed information on laboratory tests, were carefully standardized, and maintained a high quality of procedures (e.g., 3 measurements of blood pressure).

## Conclusion

MHO was not associated with a high risk of CKD and that being MANW or MAO increases the incidence of CKD. The status of metabolic health is associated more with the development of CKD than obesity. The results of this study can be used as basic data to develop programs for metabolic syndrome management, obesity prevention, and CKD prevention, and to establish governmental health policies for public health centers and clinics in Korea.

### Ethical considerations

Ethical issues (Including plagiarism, informed consent, misconduct, data fabrication and/or falsification, double publication and/or submission, redundancy, etc.) have been completely observed by the authors.
